# The effect of modifying grinding aids with PCE on grinding efficiency and powder flowability

**DOI:** 10.1038/s41598-026-46801-6

**Published:** 2026-05-05

**Authors:** Yahya Kaya, Veysel Kobya, Ali Mardani, Kambiz Ramyar

**Affiliations:** 1 Department of Civil Engineering, Faculty of Engineering, Bursa Uludag University, Bursa, Turkey; 2 Department of Civil Engineering, Ege University, Izmir, Turkey

**Keywords:** Grinding aid, Polycarboxylate ether, Hybrid admixture, Grinding efficiency, Particle size distribution, Powder fluidity, Chemistry, Engineering, Materials science

## Abstract

Cement grinding is an energy-intensive process, and while grinding aids (GAs) are common, research into hybrid systems combining different chemical mechanisms remains limited. This study evaluates the efficiency of hybrid GAs formulated by blending polycarboxylate ether (PCE) with traditional alkanolamines—triisopropanolamine (TIPA) and diethanolisopropanolamine (DEIPA)—and diethylene glycol (DEG). These formulations were tested against pure admixtures during the laboratory-scale ball milling of CEM I 42.5R cement at dosages of 0.05% and 0.1%. Results indicate that hybrid admixtures significantly outperform pure systems in reducing energy consumption. The P-DEIPA (PCE and DEIPA blend) emerged as the most efficient, achieving up to 14.4% energy savings compared to the control. Particle size distribution analysis suggests a synergistic effect: PCE prevents agglomeration while alkanolamines enhance fine-particle production, resulting in a high fraction (76.4%) of particles in the 0–32 μm range for P-DEIPA. Flowability indices—including the Carr index and angle of repose—showed improved powder characteristics across all treated samples, with the P–TIPA hybrid showing the most significant enhancement. Regarding mechanical performance, while pure TIPA yielded the highest compressive strengths (40.5 MPa at early age; 52.0 MPa at late age), the strength development of hybrid systems was influenced by a complex balance of physical refinement and hydration chemistry. These findings highlight the potential of hybrid formulations to optimize both grinding efficiency and cement performance.

## Introduction

The growing impact of the global climate crisis has placed the development of sustainable solutions and the enhancement of the efficiency of existing production processes in greenhouse gas–emitting sectors at the forefront of scientific and industrial research. Within this framework, two principal strategies have emerged in the cement industry. The first involves increasing the utilization rate of alternative binder materials to partially replace cement^1–3^, while the second focuses on the effective use of grinding aids (GA) to reduce energy consumption during cement production^4–6^.

Grinding aids are predominantly organic compounds containing alcohol or amine functional groups. During grinding, they interact with ions on the clinker surface, thereby reducing surface energy and weakening polar interactions^7,8^. This mechanism decreases interparticle cohesion, keeps the surfaces of the grinding media cleaner, and consequently enhances crushing and grinding efficiency^9,10^. Moreover, these additives have been reported to lower the energy required for agglomerate formation due to the monolayer adsorption they form on clinker surfaces^11,12^.

Polycarboxylate ether (PCE)–based GAs are distinguished by their comb-like molecular architecture. Owing to the presence of carboxylate groups along the main chain and polyethylene glycol segments in the side chains, they effectively inhibit particle agglomeration by simultaneously providing electrostatic and steric repulsion^13–16^. These superior dispersion characteristics have established PCEs as a widely adopted alternative to conventional commercial additives, and their application in the clinker grinding stage has become increasingly prevalent^11,17–19^.

Previous studies have shown that PCE, when used at approximately 0.1% of the feed rate, delivers grinding performance comparable to that of commonly employed additives such as triethanolamine (TEA) or triisopropanolamine (TIPA), while also providing improved dispersion in cement pastes^11,20^. Nevertheless, it has also been reported that the tendency of PCE molecules to complex with Ca²⁺ ions can retard hydration by altering the ionic concentration of the pore solution and partially restricting water access to cement particles^11,18,21,22^. This potential drawback necessitates careful consideration of PCEs as sole GA components, particularly regarding their effects on cement hydration kinetics.

To overcome these limitations, recent studies have focused on modifying PCEs with amine-based GA components^23–25^. The high adsorption affinity and ion-solubilizing capacity of alkanolamines can counterbalance the hydration-retarding effect of PCE^26,27^, thereby enabling a more stable and optimized hydration process^18,28^.

In the evaluation of the grinding efficiency provided by GA, the flow characteristics of the resulting cement powder constitute an additional critical parameter. The Carr index, a commonly used indicator of powder flowability, quantifies a material’s compressibility and is directly related to the Hausner ratio. Low compressibility is generally associated with enhanced flowability, whereas the Hausner ratio provides complementary insight into the cohesive behavior and flow potential of powders^29^.

The improvement in the flow performance of ground cement can be attributed to several mechanisms: (i) the abrasion of large, angular particles during grinding, leading to a more rounded particle morphology; (ii) the adsorption of GA molecules onto the surfaces of fine particles, forming surface layers that reduce interparticle agglomeration; and (iii) the narrowing of the particle size distribution induced by the use of GA^17^. However, excessive narrowing of the size distribution may also reduce flowability by increasing interparticle cohesion. Consequently, in certain cases, relatively coarser particles may exhibit superior flow behavior^17^.

The fundamental factors governing particle size’s influence on flow properties are particle surface morphology and interparticle interaction forces^30^. While sharp and rough surfaces adversely affect powder flow, smoother, more rounded particles produced with GA can improve overall flowability by compensating for the increased cohesion that may arise from a narrower particle size distribution^17,31^.

GA can also positively influence the parameters characterizing powder fluidity. For instance, Zhang et al.^17^ reported that polymer-based GA significantly reduced the angle of repose of cement powder, thereby facilitating easier particle flow. Similarly, a reduction in the angle of repose has been observed with the use of glycerol- or TEA-based admixtures^32^. These findings indicate that GA not only enhances the energy efficiency of the grinding process but also improves the flow characteristics of the final product.

In the existing literature, the effects of commercial amine- and glycol-based GA, as well as PCE-based GA, on grinding efficiency and cement properties have been extensively investigated as separate systems. However, studies addressing the performance of PCE-based hybrid GA formed by combining these two categories of admixtures remain scarce. In particular, the influence of such a hybrid GA on grinding efficiency, particle size distribution, powder flowability, and strength development has not yet been sufficiently clarified.

Within this framework, three different categories of GA were comparatively evaluated in the present study: (i) commercial alkanolamine-based admixtures TIPA, DEG, and DEIPA, whose effectiveness has been demonstrated in previous studies; (ii) a synthesized PCE-based GA; and (iii) hybrid GA obtained by the physical blending of the PCE-based GA with the commercial admixtures (P–TIPA, P–DEIPA, and P–DEG). Grinding experiments were carried out using all GA at dosages of 0.05% and 0.1% by mass of total solids.

The effects of these GA on the grinding process, cement zeta potential, particle size distribution, powder flow properties, and compressive strength development were investigated in detail.

## Materıals and methods

### Materials

In this study, three different additive systems based on distinct approaches were evaluated as GA. In the first approach, triisopropanolamine (TIPA), diethylene glycol (DEG), and diethanolisopropanolamine (DEIPA) were selected from among commercial GAs whose effectiveness had been demonstrated in the authors’ previous studies^17,28,33,34^. In the second approach, a PCE-based water-reducing admixture was synthesized and used directly as a GA. In the third approach, a novel hybrid GA system was developed by physically blending the synthesized PCE with the commercial GA. Detailed information regarding the synthesis and physical mixing procedures is provided in the Methods section.

The cements used in the experimental program were produced by grinding a mixture consisting of 96% clinker and 4% gypsum in a laboratory-scale ball mill until the targeted specific surface area (3900 ± 100 cm²/g) was achieved. It was verified that the resulting cements satisfied the requirements of the CEM I 42.5R class specified in the TS EN 197-1 standard. The chemical composition of the clinker and gypsum employed in this study is identical to that of the raw materials used in the research group’s previous investigations, and the corresponding data are presented in Table 1^6,21^.

During the grinding process, GA dosages were applied at levels of 0.05% and 0.1% by mass of the total clinker + gypsum, in accordance with dosage ranges reported in similar studies in the literature^18,19^. Within the scope of the study, 22 CEM I 42.5R cement samples were prepared and tested, including a control sample prepared without GA addition.

**Table 1:** Certain properties of clinker and gypsum.

İtem (%)															
	SiO_2_	Al_2_O_3_	Fe_2_O_3_	CaO	MgO	SO_3_	Na_2_O_eq_	Cl	Combined Water (T < 230⁰)	Other. %	C_3_S	C_2_S	C_3_A	C_4_AF	Loss of ignition
Clinker	21.52	5.43	3.31	65.38	1.04	0.38	0.83	0.01	–	1.58	56.51	19.06	8.79	10.07	0.52
Gypsum	4.98	1.21	0.83	28.94	0.83	39.67	0.37	–	18.93	4.24					

### Method

#### Synthesis method

**Fig. 1:**
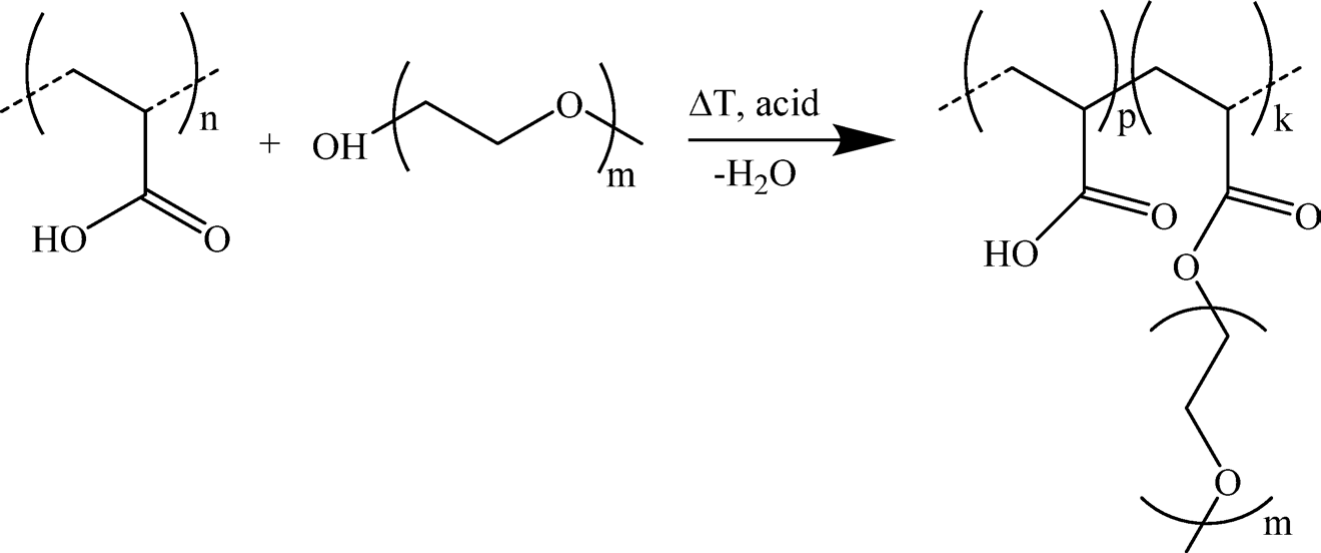
Schematic representation of the PCE synthesis via esterification between polyacrylic acid (PAA) and methyl–polyethylene glycol (mPEG) monomers.

In this study, polyacrylic acid and carboxylic acid components were used in conjunction with methyl–polyethylene glycol (mPEG) as the alcohol source for the synthesis of a PCE. The polymerization was carried out at approximately 120 °C via an acid-catalyzed esterification reaction using sulfuric acid as the catalyst. The water generated during the reaction was continuously removed from the system under vacuum. The total reaction time was set to 3 h and was terminated once the evolution of water ceased (Fig. 1).

After the reaction was completed, the product was cooled to 80 °C and subsequently diluted with water to obtain a PCE-based GA with a target solid content of 30%. The fundamental structural characteristics of the synthesized GA, including molecular weight distribution and polydispersity index (PDI), were determined by gel permeation chromatography (GPC)^35^.

According to the characterization results, the synthesized PCE-based GA exhibited a solid content of 30.12%, a pH of 3.54, a viscosity of 172 cps, and a density of 1.078 g/mL. The weight-average molecular weight (Mw) of the polymer was calculated as 37,000 Da, and the polydispersity index (Mw/Mn) was 2.1. The main-chain molecular weight was determined from the ratio of the polymer backbone contribution to the total Mw, yielding a value of 4000 Da. The side-chain molecular weight was estimated from the number-average contribution of grafted side chains per main chain, yielding 2400 Da. Consequently, the side–chain–to–main–chain length ratio was calculated to be 14, based on the total number of side chains per main-chain unit and their relative molecular weights. In other words, the ratio reflects the average number of side chains attached to each polymer backbone segment, weighted by their respective molecular weights, providing insight into the steric hindrance and dispersion efficiency imparted by the PCE-based GA.

The characteristic properties of the synthesized PCE are summarized in Table 2.

**Table 2:** The characteristic properties of the synthesized PCE.

Properties	Solid content (%)	pH	Density (g/cm^3^)	Mw* (g/mol)	PDI*** (Mw/Mn**)	Backbone length (g/mol)	Side-chain length (g/mol)	Side chain/ backbone ratio
PCE	30.12	3.54	1.078	37,000	2.1	4,000	2,400	14

*Weight average molecular weight **Number average molecular weight ***Polydispersity index.

#### Clinker grinding method

Clinker grinding experiments were conducted in a laboratory-scale Bond mill with a capacity of 5 kg and a motor power of 1.5 kW, featuring geometric and operational characteristics defined by Bond^36^. The performance of the TIPA-based grinding aids was evaluated based on grinding time and the energy required to achieve the target Blaine specific surface area (3900 ± 100 cm²/g).

Operational parameters such as ball size distribution, mill rotational speed, and feed rate play a decisive role in grinding efficiency. These parameters were determined through preliminary optimization studies and subsequently selected in accordance with the standard Bond test procedure^10^.

TIPA was dosed at levels of 0.05% and 0.1% by mass of the total clinker and gypsum. The resulting cement samples were designated according to the type and dosage of TIPA used; for example, the sample containing 0.05% TIPA was labeled “TIPA-0.05.” The specific energy consumption during the grinding process was calculated using Eq. (1).

1 $${{\mathrm{E}}_{\mathrm{g}}}={\text{ }}({\mathrm{22}}0 \times {{\mathrm{T}}_{\mathrm{g}}} \times {\mathrm{A}} \times {\mathrm{1}}000){\text{ }}/{\text{ }}({\mathrm{m}} \times {{\mathrm{T}}_{\mathrm{d}}})$$

Here, E_g_ denotes the grinding energy (kWh/ton), T_g_ is the grinding time (hours), A is the electric current, m is the feed mass (kg), and Td is the mill factor, taken as a constant of 4 according to the manufacturer’s specifications.

### Particle size distribution and zeta potential

The particle size distribution (PSD) of the cement samples was determined using a Malvern Mastersizer 2000 laser diffraction analyzer equipped with a Hydro 2000 S wet dispersion unit. To prevent agglomeration and to ensure a homogeneous dispersion, each sample was subjected to ultrasonic treatment for 10 min before analysis.

The zeta potential (ZP) values of the cement samples were measured using a Zetasizer ZS90 instrument. A solid-to-liquid ratio of 0.1 g/mL was employed; the suspension pH was adjusted to 12.5, and 0.01 M KCl was u.

sed as the background electrolyte to maintain a controlled ionic environment. All measurements were conducted at 23 °C, and the applied electric field was set to 50–100 V. The zeta potential measurements were performed according to standard procedures reported in the literature.

### Calculation of the n value according to the Rosin–Rammler distribution

The Rosin–Rammler distribution model was employed to characterize the particle size distribution. This model enables the PSD to be expressed as a continuous function and provides quantitative information on powder fineness and distribution homogeneity. In the present study, the parameter $\:n$(the slope coefficient of the distribution) for each cement sample was calculated using Eq. (2).

2 $${\mathrm{R}}\,=\,{\mathrm{exp}}~\left[ { - {{\left( {\frac{x}{{{x^1}}}} \right)}^n}} \right]$$

Here, R denotes the mass fraction remaining above a given particle size; X represents the particle size (µm); x^1^ is the characteristic size, defined as the particle size at which 63.2% of the material passes; and $\:n\:is$ the slope parameter of the distribution.

### Powder flowability tests

To evaluate the flow properties of the produced cements, fundamental powder flow parameters—including bulk density, tapped density, Hausner ratio, Carr index, and angle of repose—were determined. These measurements were performed in accordance with the detailed standard procedures previously published by the research group^21^.

### Compressive strength

Mortar mixtures were prepared according to the proportions and procedures specified in ASTM C109 and homogenized using a Hobart-type laboratory mixer. The spread diameter of the fresh mortars was measured in accordance with ASTM C1437, and the value was maintained within 220 ± 20 mm for all mixtures.

Compressive strength tests were conducted in accordance with ASTM C109. For each mixture, the compressive strength was calculated as the average of three replicate specimens prepared under identical conditions.

## Results and discussions

### Chemical characterization

**Fig. 2:**
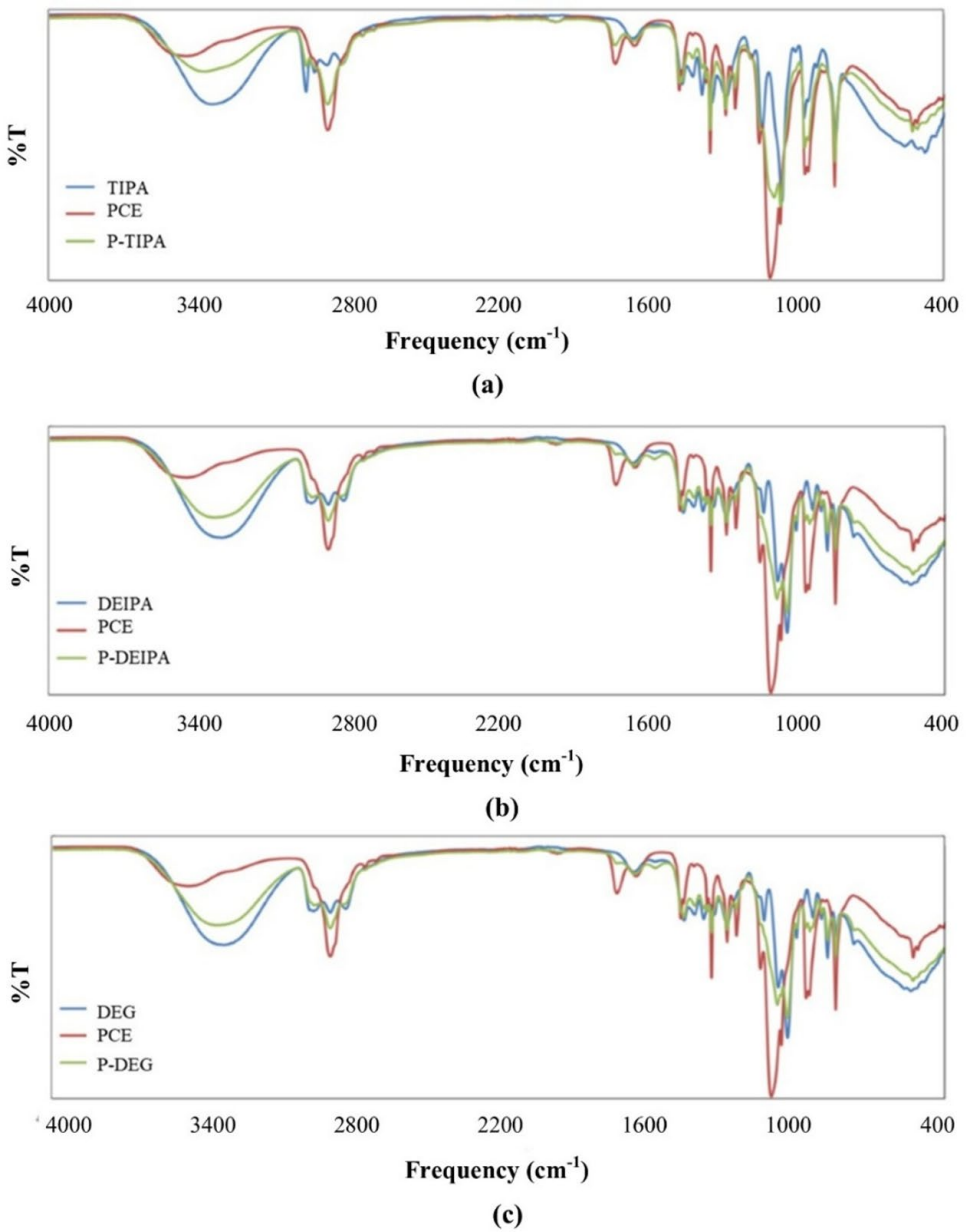
FTIR spectra of synthesized PCE-based modified GAs.

Fourier Transform Infrared (FTIR) spectroscopy analysis was performed to verify the chemical structures of the synthesized PCE and PCE-based hybrid dopants. The FTIR spectra of all synthesized dopants are presented comparatively in Fig. 2.

An examination of the spectra in Fig. 2 reveals that the broad bands observed at approximately 3300, 1630, and 550 cm⁻¹ arise from the stretching and bending vibrations of both adsorbed water and hydrogen-bonded OH groups. The characteristic peaks associated with the synthesized PCE structure can be summarized as follows: the bands in the range of 2934–2877 cm⁻¹ are attributed to aliphatic C–H stretching vibrations, whereas the weak bands in the range of 1455–1253 cm⁻¹ correspond to C–H bending vibrations. The presence of ether linkages (C–O–C) is confirmed by the distinct and sharp C–O stretching band observed at approximately 1100 cm⁻¹.

### Grinding efficiency

**Fig. 3:**
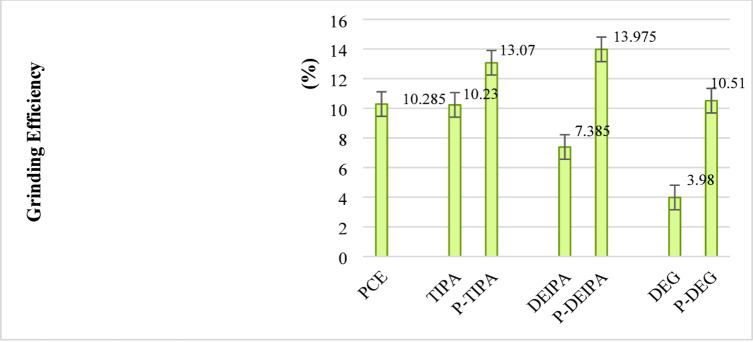
Average grinding efficiencies of the GAs used.

The effect of new-generation PCE-based GAs on grinding efficiency was evaluated based on the specific energy required to achieve the target Blaine surface area (3900 ± 100 cm²/g). The energy consumption values obtained from the grinding experiments, along with their corresponding relative energy efficiencies, are presented in Table 3. The average increases in energy efficiency provided by the GA are illustrated in Fig. 3.

**Table 3:** Energy values and relative energy efficiencies in the grinding process.

	Blaine (cm^2^/g)	Number of revolutions required to achieve the target Blaine	Grinding time for target Blaine (min.)	Consumed energy (kWh)	Relative Energy efficiency (%)
Control	3940	8800	125.7	52.78	
PCE-0.05	3982	7720	110.3	46.30	11.77
PCE-0.1	3902	7980	114.0	47.86	8.80
TIPA-0.5	3945	7900	112.9	47.38	10.23
TIPA-0.1	3906	7900	112.9	47.38	10.23
P-TIPA-0.5	3820	7620	108.9	45.70	13.41
P-TIPA-0.1	3860	7680	109.7	46.06	12.73
DEIPA-0.05	3905	8100	115.7	48.58	7.95
DEIPA-0.1	3990	8200	117.1	49.18	6.82
P-DEIPA-0.05	3812	7530	107.6	45.16	14.43
P-DEIPA-0.1	3867	7610	108.7	45.64	13.52
DEG-0.05	3860	8450	120.7	50.68	3.98
DEG-0.1	3850	8450	120.7	50.68	3.98
P-DEG-0.05	3910	7750	110.7	46.48	11.93
P-DEG-0.1	3925	8000	114.3	47.98	9.09

According to the experimental results, achieving the target Blaine surface area for the control cement (GA-free) required 8800 revolutions, 125.7 min, and 52.78 kWh of energy. When used alone, the synthesized PCE reduced the energy consumption to 46.30–47.86 kWh, corresponding to an efficiency increase of 8.80–11.77%. The TIPA-based GA exhibited a comparable level of improvement, with an average efficiency gain of 10.23%. Lower efficiency increases were recorded for DEIPA (6.82–7.95%) and, in particular, for DEG (3.98%).

In contrast, the hybrid GA formulations (P–TIPA, P–DEIPA, and P–DEG), prepared by physically blending PCE with the commercial GA at a 1:1 mass ratio, yielded the lowest energy consumption values. Among these systems, the P–DEIPA blend achieved the highest relative energy efficiency, ranging from 13.52% to 14.43%, reducing the energy demand to 45.16–45.64 kWh. The incorporation of GA markedly decreased the number of revolutions, grinding time, and, consequently, the energy required to attain the target fineness.

These improvements can be attributed to the ability of GA to (i) inhibit the re-agglomeration of newly formed fine particles, (ii) reduce coating on the grinding media and mill lining, and (iii) enhance powder flowability, thereby minimizing inefficient friction and energy losses within the mill^19,33,37,38^. These effects are fundamentally associated with the adsorption of GA molecules onto the clinker grain surfaces^7,12,37^.

Glycol-derivative GA (e.g., DEG) reduces interparticle cohesion by adhering to particle surfaces through hydroxyl groups; however, their ionic interaction and complexation capacities are more limited than those of alkanolamines. Consequently, although glycols provide a measurable increase in energy efficiency, their optimum dosage range is relatively narrow, and their overall effect tends to be weaker^4,34,39^. The higher efficiencies observed for alkanolamine-based GA, such as TIPA and DEIPA, are attributed to their more effective surface adsorption, arising from the combined presence of polar hydroxyl groups and amine functionalities, as well as their ability to interact with Ca²⁺ ions^35^.

Irrespective of GA type, all additives increased grinding efficiency by 4% to 14%. The performance ranking of the hybrid GA obtained by modifying commercial GA with PCE, from highest to lowest, was P–DEIPA, P–DEG, and P–TIPA.

**Fig. 4:**
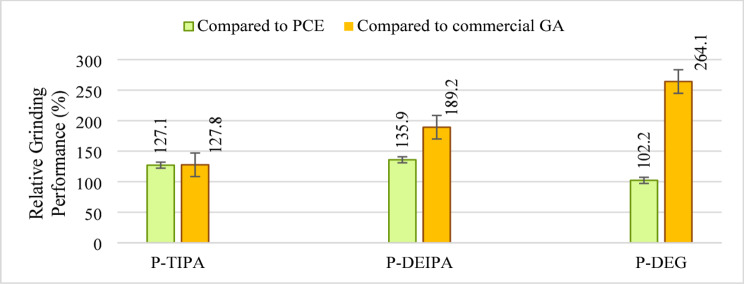
Improvements in the grinding performance of hybrid GA relative to commercial GA and PCE, evaluated in terms of synergistic effects.

To more clearly elucidate the synergistic effect of these hybrid systems, the grinding performances of the PCE-based GA (P–TIPA, P–DEIPA, and P–DEG) are presented comparatively in Fig. 4, alongside those of pure PCE and the corresponding commercial GA (TIPA, DEIPA, and DEG).

The comparison presented in Fig. 4 indicates that all PCE-based hybrid GA outperform both their corresponding commercial GAs and pure PCE. For instance, the P–DEG formulation enhanced grinding performance by approximately 1.5 times relative to DEG, while exhibiting no significant difference compared with pure PCE. The P–DEIPA system increased performance by about 1.1 times compared with DEIPA and by 28% relative to pure PCE. Similarly, P–TIPA achieved 36% improvement over the TIPA-based GA and 24% over pure PCE.

**Fig. 5:**
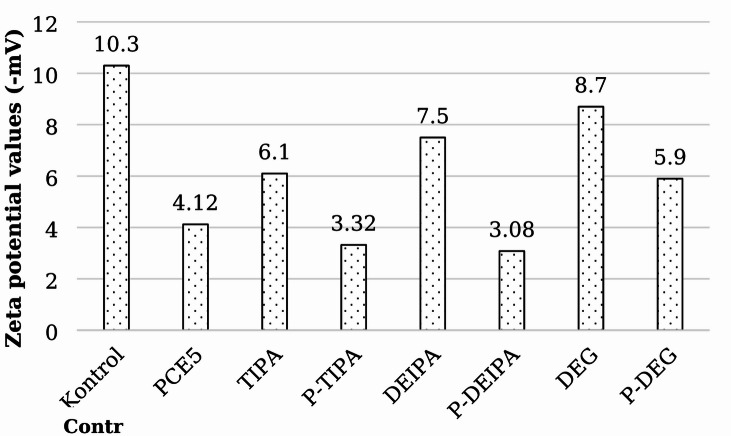
Zeta potential values of control cements without GA and cements containing GA at a dosage of 0.05%.

**Fig. 6:**
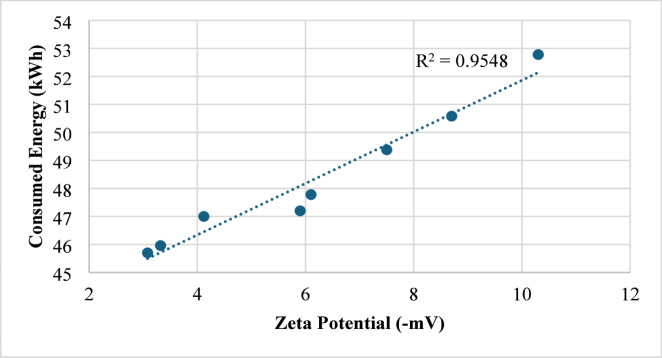
The relationship between energy consumed in grinding and Zeta potential values.

These findings suggest that the lower the intrinsic performance of the initial commercial GA, the more pronounced the performance enhancement achieved through PCE modification. To elucidate the surface interaction mechanisms of GA and to relate them to grinding performance, the zeta potential values of the control sample and the cements containing 0.05% GA are presented in Fig. 5. The relationship between the energy consumed during grinding and the corresponding zeta potential is illustrated in Fig. 6.

An examination of Fig. 5 shows that, in cements incorporating PCE-based hybrid GA (P–TIPA, P–DEIPA, and P–DEG), the zeta potential values approach zero irrespective of the admixture type. A near-zero zeta potential indicates that the net electrostatic charge on the particle surfaces has been neutralized and that a charge balance has been established^10,40^. In dry grinding systems, clinker particles can accumulate electrostatic charges due to friction and particle–particle interactions during milling. The build-up of these surface charges increases interparticle attraction, promoting agglomeration and coating of grinding media and mill liners. When the zeta potential approaches zero, the electrostatic imbalance between particles is reduced, which diminishes electrostatic attraction and limits particle agglomeration. As a result, particles remain more dispersed during grinding, facilitating more effective breakage and improving grinding efficiency. Furthermore, as illustrated in Fig. 6, a clear relationship exists between the energy consumed during grinding and the zeta potential. This correlation supports the hypothesis that GA directly influences grinding efficiency by neutralizing particle surface charges^19^.

Indeed, whereas the control sample consumed the highest grinding energy, the lowest zeta potential and the lowest energy consumption were observed for the cement containing the P–DEIPA admixture. This observation is consistent with the superior grinding efficiency of P–DEIPA reported in Table 3; Fig. 3.

The enhanced performance observed for the hybrid GA formulations (P–TIPA, P–DEIPA, and P–DEG), obtained by physically blending PCE with commercial GA, is attributed to the synergistic action of two distinct mechanisms. These mechanisms comprise (i) the strong adsorption of PCE onto clinker surfaces and the steric hindrance effect imparted by its long side chains^18,21,41^, and (ii) the surface energy–lowering and ionic interaction–modifying roles of alkanolamine- or glycol-derivative GA.

### Particle size distribution

The results of the particle size distribution analyses of the produced cements are presented in Table 4. The table reports the d10, d50, and d90 particle size values for each sample, along with the parameter $\:n$, which represents the slope of the Rosin–Rammler distribution curve.

**Table 4:** Particle size distribution and some properties of cements.

	0–10 μm	11–32 μm	33–45 μm	46–60 μm	61–90 μm	91- µm	D10 µm	D50 µm	D90 µm	*n*	0–32 μm
Control	36.15	31.15	11.08	6.21	6.06	9.35	2.28	19	78	0.804	67.3
PCE-0.05	39.52	32.28	12.21	6.99	5.69	3.31	2.41	18.05	48.31	0.917	71.8
PCE-0.1	39.777	33.502	11.913	6.429	5.321	3.059	2.39	18.73	44.81	0.931	73.279
TIPA-0.05	40.51	33.11	12.07	6.36	5.22	2.69	1.99	15.9	59	0.931	73.62
TIPA-0.1	41.8	31.33	11.79	6.67	5.92	2.48	1.8	15.4	60.7	0.900	73.13
P-TIPA-0.5	41.2	33.78	12.35	5.36	4.86	2.45	1.75	14.8	63.5	0.946	74.98
P-TIPA-0.1	41.67	33.85	13.05	5.01	4.22	2.2	1.7	14.5	64	0.963	75.52
DEIPA-0.05	39.42	32.41	11.33	5.95	4.96	5.93	2.01	16.5	68.2	0.863	71.83
DEIPA-0.1	40.15	31.19	11.75	6.73	6.04	4.12	1.93	16.3	65.5	0.878	71.34
P-DEIPA-0.05	41.63	34.81	12.12	5.52	3.91	2.01	1.85	15.2	66	0.972213	76.44
P-DEIPA-0.1	41.96	34.32	12.21	5.11	4.22	2.18	1.8	15	65.1	0.95832	76.28
DEG-0.05	36.04	32.48	13.51	8.16	7.22	2.56	2.2	18.8	64.6	0.966	68.52
DEG-0.1	37.7	34.11	13.41	7.42	5.38	1.98	2.19	18.5	56.4	0.992	71.81
P-DEG-0.05	37.25	34.74	12.87	7.55	5.18	2.41	2.1	18	58.5	0.989597	71.99
P-DEG-0.1	38.47	33.78	12.57	8.1	4.84	2.24	2.05	18.1	57	0.974482	72.25

The PSD of the control cement indicates that agglomeration is not effectively suppressed during the grinding process and, in particular, that coarse particles are not sufficiently broken down. This is evidenced by the relatively high fraction above 91 μm, which remains at 9.35%. This result suggests that the fine particles generated during grinding tend to recombine into large agglomerates, which persist unbroken within the mill environment. In parallel, the high d90 value (78 μm) and the relatively low Rosin–Rammler slope parameter (*n* = 0.804) indicate that particle sizes are distributed over a wide range and exhibit a non-homogeneous distribution.

When PCE is used as a GA, the PSD’s character changes markedly. In cements containing PCE, a pronounced reduction in the coarse tail fraction is observed. The decrease in the fraction above 91 μm to approximately 3% and the reduction in the d90 value to 44–48 μm demonstrate that PCE effectively inhibits the re-agglomeration of coarse particles during grinding. This behavior can be attributed to the adsorption of PCE onto particle surfaces owing to its polymeric structure, together with the steric repulsive forces generated by its side chains^42^. The literature further emphasizes that side-chain length and density play a decisive role in the dispersion capability of PCE and, consequently, in grinding efficiency^43^. The rapid coating of newly formed surfaces by PCE molecules within the grinding environment weakens van der Waals attractive forces, thereby preventing fine particles from recombining and forming coarse agglomerates^19^.

Overall, the grinding mechanism in PCE-containing systems is fundamentally governed by agglomeration control and coarse particle elimination. Although the increase in fine fractions within the 0–32 μm range remains relatively limited with PCE addition, the enhanced distribution homogeneity and the increase in the n value indicate that the grinding process becomes more controlled and efficient.

In contrast, the PSD of cements containing TIPA indicates a different grinding mechanism than that observed in PCE-based systems. The substantial increase in the 0–10 μm and 11–32 μm fractions, together with the reduction of the d50 value to approximately 15–16 μm, indicates that TIPA strongly promotes fine particle generation during grinding. However, the fact that the d90 values remain higher than those obtained with PCE reveals that coarse particles are not fully controlled. This suggests that the grinding mechanism of TIPA, unlike that of PCE, primarily facilitates particle fracture by reducing surface energy and promoting microcrack propagation^21,42^.

The adsorption of TIPA molecules onto mineral surfaces via hydroxyl and amine functional groups lowers surface energy, leading to more effective fragmentation, particularly within the medium- and fine-particle size ranges^21^. Nevertheless, because TIPA does not form a steric barrier, the recombination of ultrafine particles cannot be prevented entirely, and coarse-tail suppression is therefore less effective than in PCE-containing systems. Consequently, the grinding mechanism of TIPA can be described as one that “enhances fine fraction production but provides limited control over the coarse tail.

The PSD profile obtained in systems where PCE and TIPA are physically mixed indicates that two distinct grinding mechanisms are activated simultaneously. In these mixtures, the 0–10 μm and 11–32 μm fractions reach their highest levels, while the fraction above 91 μm decreases to approximately 2–2.5%. At the same time, the d10 and d50 values attain their lowest levels, and the Rosin–Rammler slope parameter (n) increases markedly. These findings demonstrate that the steric, agglomeration-suppressing effect of PCE and the fracture-facilitating effect of TIPA, arising from reduced surface energy, operate synergistically.

During grinding, TIPA promotes the formation of new surfaces and fine fragmentation, whereas PCE inhibits the re-agglomeration of these newly formed fine particles, ensuring their retention in the system. Consequently, PCE+TIPA mixtures provide the most balanced and effective PSD modification in terms of both fine fraction production and coarse-tail suppression. This outcome shows that physical mixing alone can generate a strong synergy through grinding physics, without requiring a specific chemical interaction between the components^42^.

The PSD observed in systems containing DEIPA tends to be similar to that of TIPA, although the effect remains more limited. When DEIPA is used alone, an increase in the fine fraction within the 0–32 μm range is observed, while the proportion of coarse particles above 91 μm remains relatively high at certain dosages. This behavior indicates that DEIPA’s ability to reduce particle surface energy and suppress agglomeration is weaker than that of TIPA. This interpretation is consistent with the measured zeta potential values of cement particles containing DEIPA. Moreover, the fact that the d50 values are lower than those of the control sample but higher than those of the TIPA-containing samples further supports this assessment. Overall, the grinding mechanism of DEIPA can be described as intermediate: it promotes fine particle formation but provides only limited control over agglomeration.

In hybrid systems formed by the physical mixing of PCE and DEIPA, the strongest synergistic effect on PSD modification is observed. In these mixtures, the proportion of fine fractions (0–32 μm) reaches approximately 76%, while the coarse fraction (> 91 μm) decreases to about 2%, and the n value attains its highest levels. This behavior indicates that the steric hindrance provided by PCE, which suppresses agglomeration, and DEIPA’s role in modifying particle surface interactions and facilitating fracture act cooperatively. During grinding, DEIPA supports fine fragmentation, while PCE prevents reagglomeration of the resulting fine particles and maintains dispersion stability, leading to a fine, homogeneous particle size distribution. This synergistic mechanism explains why the PCE+DEIPA hybrid admixture yields a substantially more effective PSD improvement than DEIPA used alone.

In systems containing DEG, the grinding mechanism differs markedly from that of alkanolamine-based admixtures. DEG primarily acts by reducing surface tension, thereby lowering particle–particle friction and increasing system fluidity^19,29^. As a result of this primary function, the improvement in PSD observed in cements with DEG admixture is mainly reflected in a limited reduction of the medium and coarse fractions. The increase in the fine fraction (0–32 μm) does not reach the levels achieved with TIPA or DEIPA, and the d50 values remain close to those of the control sample. Nevertheless, the increase in the Rosin–Rammler slope parameter (n) indicates that the particle size distribution becomes more uniform and homogeneous, even though the overall fineness enhancement remains moderate.

In hybrid systems formed by the physical mixing of PCE and DEG, the agglomeration-suppressing effect of PCE and the friction-reducing property of DEG combine to produce a moderate synergy. In these mixtures, the coarse fraction above 91 μm is effectively controlled; however, the increase in fine fraction production does not reach the levels observed in the PCE+TIPA or PCE+DEIPA systems. This behavior indicates that, due to DEG’s limited capacity to promote particle fracture directly, the contribution of PCE in this hybrid system is primarily confined to coarse-tail elimination and agglomeration control.

Overall, the particle-size distribution data demonstrate that the different chemical structures of GAs influence cement grinding via distinct mechanisms. PCE-based admixtures predominantly act by suppressing agglomeration and reducing the coarse particle fraction (i.e., functioning as a “coarse-tail trimmer”). In contrast, alkanolamine-derived admixtures such as TIPA and DEIPA primarily facilitate particle fracture, thereby increasing fine-fraction production. By combining these two fundamental mechanisms—agglomeration control and fine-fragmentation promotion—through physical mixtures, it becomes possible to simultaneously support fine particle formation during grinding and prevent re-aggregation, ensuring stable dispersion. This dual action results in a pronounced performance synergy.

**Fig. 7:**
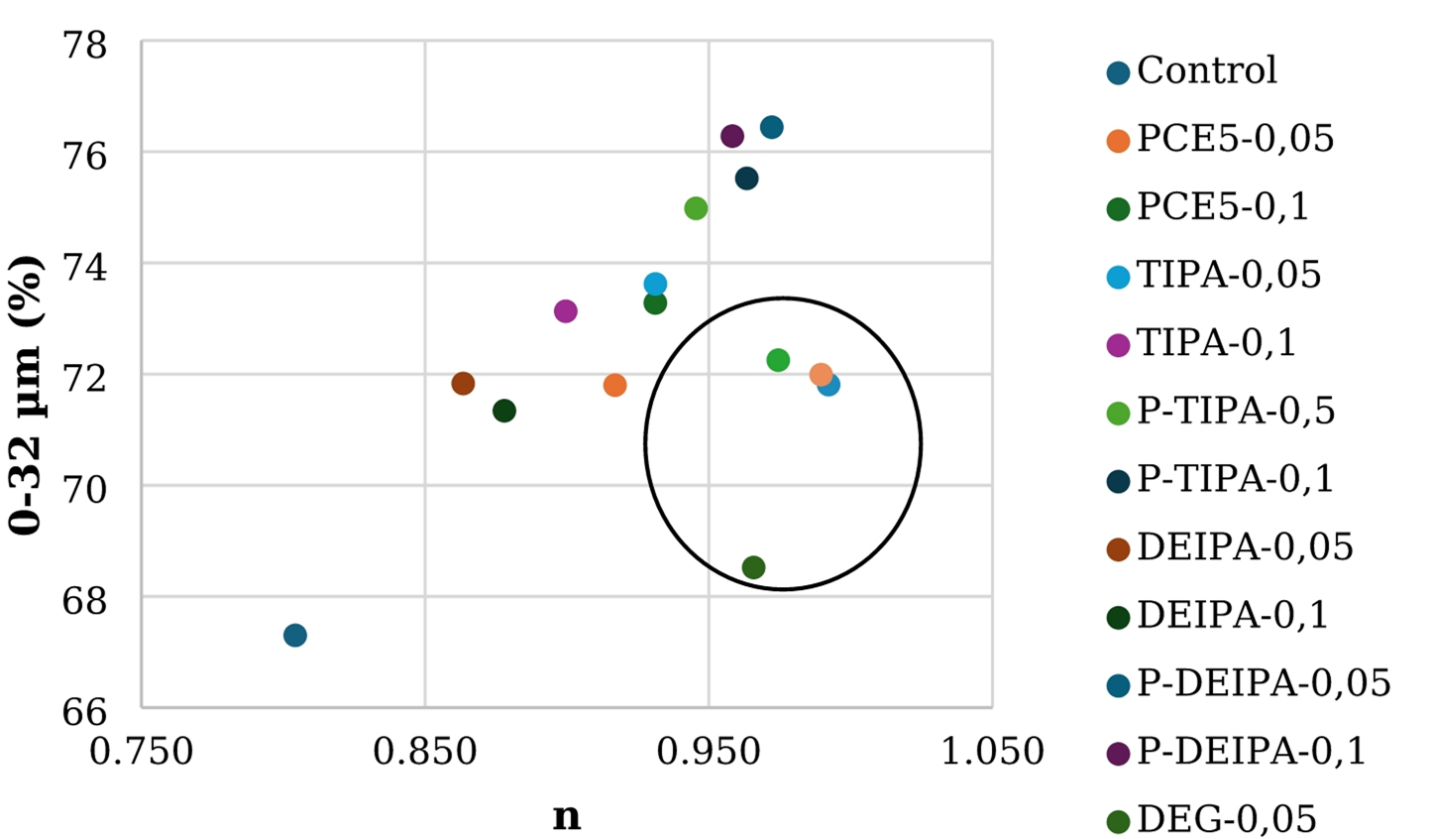
Relationship between the fine fraction of cement particles (0–32 μm) and the Rosin–Rammler slope parameter (n) of the particle size distribution curve.

Consequently, hybrid systems comprising a physical mixture of PCE and conventional GAs can be regarded as next-generation grinding facilitators that simultaneously optimize multiple aspects of the grinding process (fragmentation and dispersion) without increasing the total admixture dosage. Figure 7 illustrates the relationship between the fine particle content in the 0–32 μm range of the produced cement samples and the Rosin–Rammler slope parameter (n) of the PSD curve.

As shown in Fig. 7, a clear linear relationship is observed between the 0–32 μm fine fraction and the dispersion slope parameter (n) for all additives except those containing DEG. According to the Rosin–Rammler model, an increase in the n value does not necessarily imply a higher proportion of ultrafine particles; rather, it reflects a shift toward a steeper distribution curve, corresponding to a narrower and more homogeneous particle size range^44–46^. In this context, the elevated n values recorded in DEG-containing systems indicate that, although the fine fraction (0–10 μm) does not increase to the same extent as in alkanolamine-based GAs, the coarse tail—particularly in the 61–90 μm and > 91 μm ranges—is effectively suppressed. This behavior results in clustering of particles within a narrower size interval.

Indeed, although the d90 values of DEG-modified samples decrease markedly relative to the control, the comparatively modest changes in d10 and d50 clearly demonstrate that the particle size distribution becomes concentrated around medium sizes, with a reduced distribution width. Glycol-derived GAs such as DEG exhibit weaker and less selective adsorption onto clinker mineral surfaces than alkanolamines. Their primary function is instead to improve frictional conditions within the mill and to enhance the efficiency of energy transfer during grinding. The literature emphasizes that the dominant mechanisms of glycol-based additives include reducing coating on grinding balls and mill liners, minimizing the “cushioning” effect induced by excessive fines, and weakening re-agglomeration driven by electrostatic attraction^19,33^. These mechanisms ensure that the energy supplied to the mill is more efficiently transmitted to coarse and medium-sized particles, leading to more controlled and regular fragmentation within the targeted size range^47^.

Consequently, in DEG-containing systems, grinding proceeds not toward the generation of new, extremely fine surfaces but rather toward the controlled crushing of existing coarse particles and the stabilization of the medium-sized fraction. As a result, while the increase in the 0–32 μm fine fraction remains limited, the coarse tail fraction (especially > 61 μm) decreases, and the overall particle size distribution shifts toward a narrower range. An increase in the Rosin–Rammler slope parameter (n) quantitatively expresses this narrowing^48^. Thus, in DEG-based systems, a high n value should be interpreted as an indicator of particle size homogenization and distribution uniformity rather than as evidence of dominant fine fraction production.

In contrast, alkanolamine-based GAs, such as TIPA and DEIPA, interact more strongly with mineral surfaces via chemical interactions, facilitating microcrack initiation and propagation on newly formed surfaces during grinding. By reducing particle surface energy, these additives lower fracture resistance, thereby promoting the formation of denser fine particles, particularly in the 0–10 μm and 11–32 μm ranges. Owing to this mechanism, the 0–32 μm fine fraction increases substantially in alkanolamine-containing systems, accompanied by pronounced decreases in d10 and d50. In these systems, the increase in the Rosin–Rammler slope (n) occurs in parallel with intensified fine fraction production.

By contrast, because this dense fine fraction production mechanism is not operative in DEG-based systems, the relationship between the 0–32 μm fraction and the n value exhibits a distinctly different character from that observed for alkanolamine-based GAs. This divergent behavior underscores a crucial functional distinction among grinding aids. While amine and alkanolamine derivatives can be primarily classified as fracture facilitators and fine fraction producers, glycol-based additives are more accurately described as agents that “homogenize the particle size distribution by reducing agglomeration and coating.” In this context, the observation that DEG increases the Rosin–Rammler slope parameter (n) while inducing only a limited rise in the 0–32 μm fraction is fully consistent with the fundamental function and behavior of this class of additives as reported in the literature.

### Powder flow characteristics

The powder flow characteristics of all produced cement samples—namely bulk density, apparent density, Carr index, Hausner ratio, angle of repose, and the corresponding flowability classifications derived from these parameters—are summarized in Table 5.

**Table 5:** Cement parameters include bulk density, apparent density, Carr index, Hausner ratio, angle of view, and flow classes.

	Bulk density (kg/m^3^)	Apparent density (kg/m^3^)	Carr index	Flowability class*	Hausner rate	Flowability class**	Angle of repose (°)	Flowability class***
Control	761.24	1256.12	39.40	Extremely Poor	1.650	Extremely Poor	55.71	Very Poor
PCE-0.05	948.71	1382.6	31.38	Poor	1.457	Very Poor	45.28	Passable
PCE-0.1	912.26	1295.12	29.56	Poor	1.420	Poor	43.99	Passable
TIPA-0.05	872.56	1354.24	35.57	Very Poor	1.552	Very Poor	46.12	Poor
TIPA-0.1	897.21	1342.17	33.15	Very Poor	1.496	Very Poor	45.98	Poor
P-TIPA-0.05	976.25	1348.12	27.58	Poor	1.381	Poor	44.73	Passable
P-TIPA-0.1	982.76	1327.93	25.99	Poor	1.351	Poor	42.79	Passable
DEIPA-0.05	812.45	1297.5	37.38	Very Poor	1.597	Extremely Poor	47.42	Poor
DEIPA-0.1	901.09	1372.6	34.35	Very Poor	1.523	Very Poor	46.28	Poor
P-DEIPA-0.05	863.21	1302.24	33.71	VeryPoor	1.509	Very Poor	45.13	Passable
P-DEIPA-0.1	892.86	1278.27	30.15	Poor	1.432	Poor	43.28	Passable
DEG-0.05	812.39	1296.5	37.34	Very Poor	1.596	Extremely Poor	49.27	Poor
DEG-0.1	851.31	1325.4	35.77	Very Poor	1.557	Very Poor	46.34	Poor
P-DEG-0.05	893.02	1324	32.55	Very Poor	1.483	Very Poor	45.82	Poor
P-DEG-0.1	879.23	1276.26	31.11	Poor	1.452	Poor	44.17	Passable

* Carr Index; Excellent ≤ 10; Good :11–15; Fair: 16–20; Passable:21–25; Poor: 26–31; VeryPoor: 32–37; ExtremelyPoor ˃38 (Kaya ve ark., 2024) **HausnerRate; Excellent : 1.0–1.11; Good :1.12–1.18; Fair: 1.19–1.25; Passable:1.26–1.34; Poor: 1.35–1.45; VeryPoor: 1.46–1.59; ExtremelyPoor˃1.60 (Kaya ve ark., 2024) *** Repose angle; Excellent : 25–30; Good :31–35; Fair: 36–40; Passable:41–45; Poor: 46–55; Very Poor: 56–65; ExtremelyPoor˃66 (Katircioglu-Bayel, 2023).

### Carr index and Hausner ratio

The Carr index is an indicator of powder compressibility and is directly related to the Hausner ratio. Together, these parameters provide a practical and widely accepted means of evaluating powder flow behavior. In general, lower compressibility corresponds to higher powder fluidity. The Hausner ratio serves as a complementary parameter, offering additional insight into a powder’s flow potential and interparticle cohesion^29^.

**Fig. 8:**
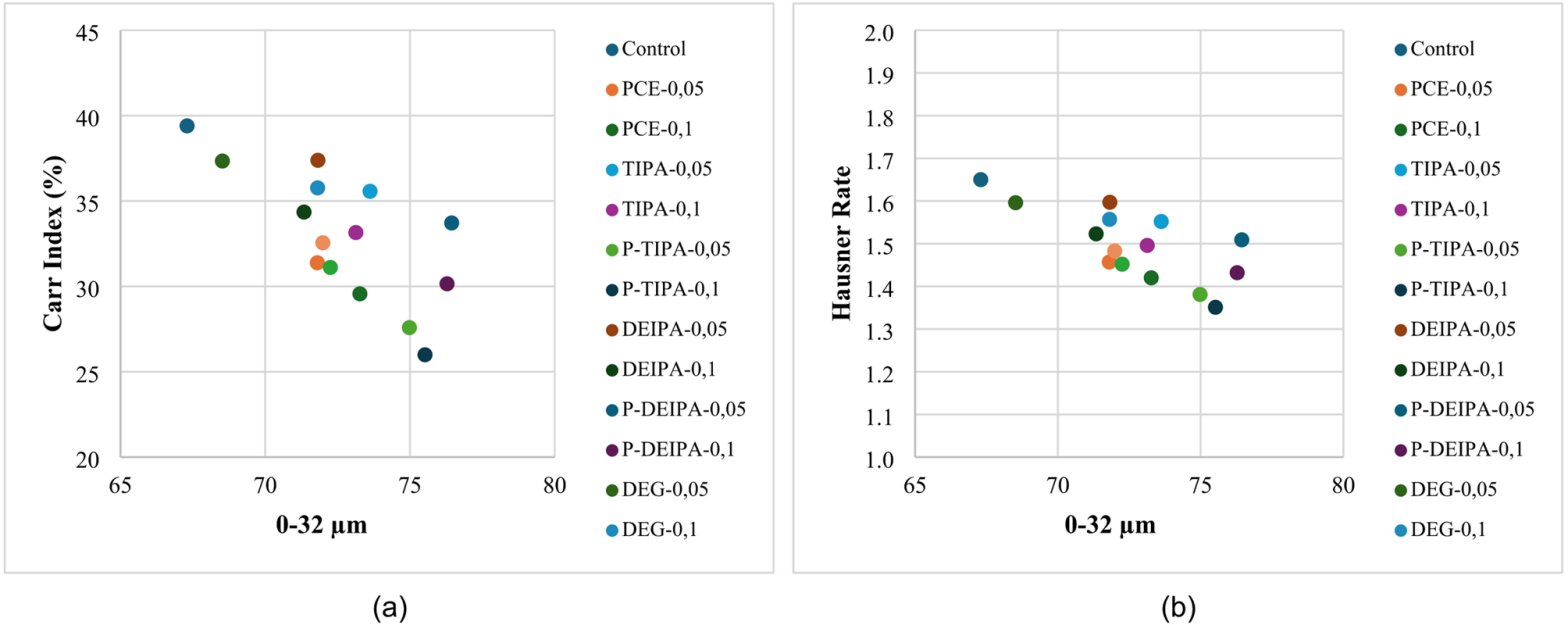
Variation of (**a**) Carr index and (**b**) Hausner ratios according to the 0–32 μm particle size fraction of cements.

In this study, the Carr index and Hausner ratio values of the cement samples were analyzed in detail in relation to the fine particle content in the 0–32 μm size range. The variations in these two flow parameters as a function of the increase in the 0–32 μm fraction for the control sample and all GA-containing cements are presented comparatively in Fig. 8.

As shown in Fig. 8, all samples containing GA, irrespective of admixture type and dosage, exhibited an increase in the 0–32 μm fine fraction relative to the control cement. In parallel, both the Carr index and Hausner ratio values decreased compared with the GA-free sample, indicating an overall improvement in powder flowability.

Based on the Carr index classification, the control cement was categorized as “Extremely Poor,” whereas cements containing TIPA-, DEIPA-, and DEG-based GA remained in the “Very Poor” class at all dosages. All samples incorporating PCE and P–TIPA GA were classified as “Poor,” while cements with P–DEIPA and P–DEG GA reached the “Poor” class only at the 0.1% dosage. At the lower dosage (0.05%), these latter systems remained within the “Very Poor” category (Table 5).

The Carr index values of cements produced with commercial GA (TIPA, DEIPA, and DEG) decreased by approximately 5–16% relative to the control sample. When pure PCE was used as the GA, the improvement increased to 20–25%. In hybrid systems formed by physically mixing PCE with commercial GA (P–TIPA, P–DEIPA, and P–DEG), the reduction in Carr index ranged from 14% to 34%. The most pronounced enhancement was observed for the P–TIPA system, which achieved a 30–34% decrease in the Carr index compared with the control cement.

The superior flow performance of PCE relative to conventional GA can be attributed to its more effective adsorption onto cement particle surfaces (Fig. 5). In addition, it has been reported that a high anionic-to-nonionic group ratio in the PCE structure enhances adsorption capacity, thereby improving dispersion and flowability^49^. The favorable powder flow characteristics of PCE are also consistent with its high grinding efficiency and the improved particle size distribution (PSD) observed for this additive (Fig. 3; Table 4).

An analysis of the Hausner ratio values revealed trends entirely consistent with the Carr index results, as expected given the direct mathematical relationship between the two parameters. According to the Hausner-based flow classification, the control, DEIPA-0.05, and DEG-0.05 samples were categorized as “Extremely Poor,” whereas TIPA-0.05, TIPA-0.1, DEIPA-0.1, DEG-0.1, PCE-0.05, P–DEIPA-0.05, and P–DEG-0.05 fell into the “Very Poor” class. Samples exhibiting improved performance and classified as “Poor” included PCE-0.1, P–TIPA-0.05, P–TIPA-0.1, P–DEIPA-0.1, and P–DEG-0.1.

This classification clearly demonstrates that PCE-modified hybrid GA substantially enhances the flow performance of conventional GA systems. These findings are in good agreement with the grinding efficiency and PSD results discussed earlier (Fig. 3; Table 4). Among all samples, those containing the P–TIPA GA exhibited the best flowability, achieving a 16–18% reduction in the Hausner ratio relative to the control cement. Overall, both the Carr index and Hausner ratio data corroborate the general trend observed throughout this study: modification of commercial GA with PCE leads to a marked and systematic improvement in grinding performance and powder flow behavior.

### Angle of repose

**Fig. 9:**
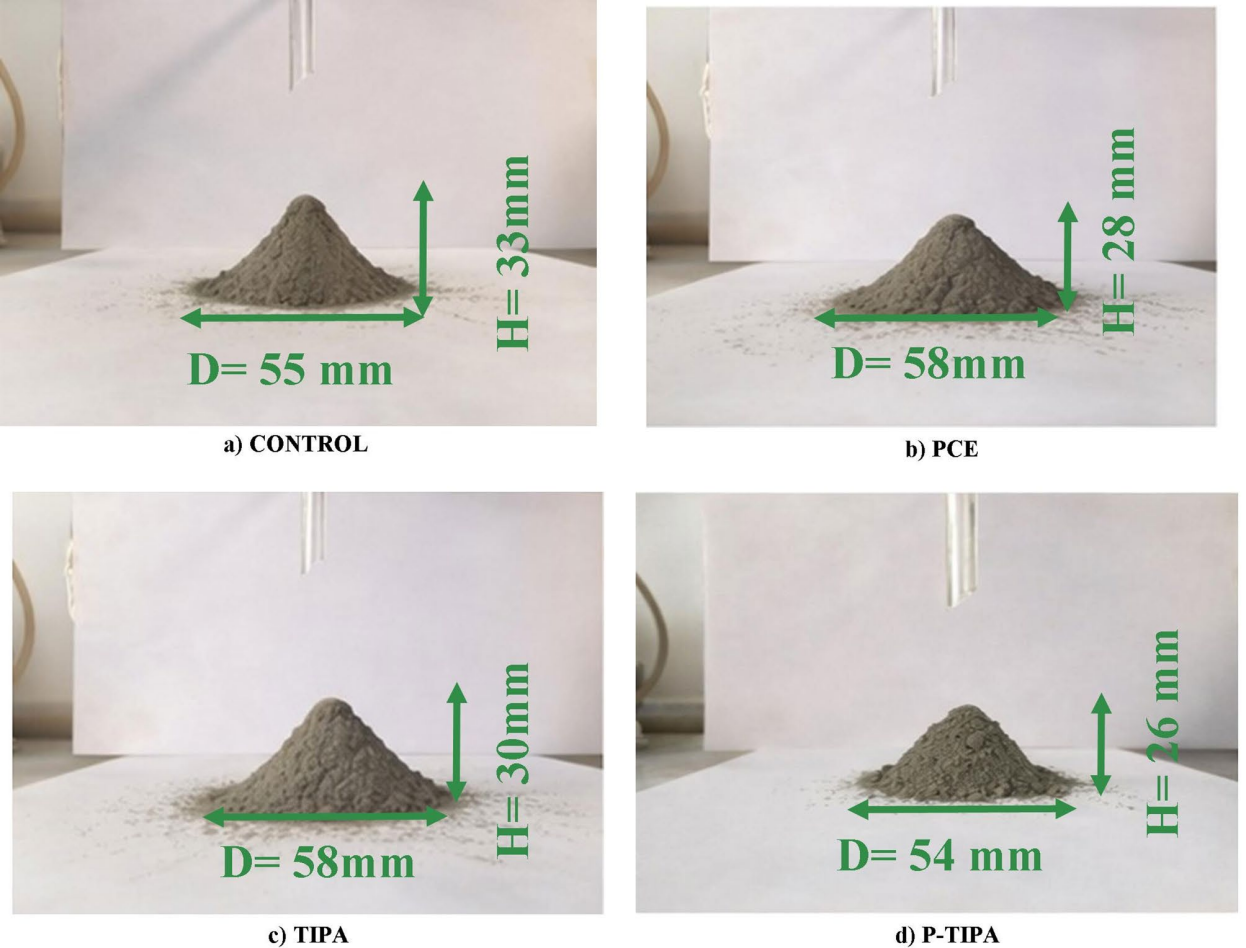
Images of the angle measurements of the selected cement samples: (**a**) Control, (**b**) PCE, (**c**) TIPA, (**d**) P-TIPA.

The angle of repose is a fundamental parameter widely used to characterize the flow behavior of cement powders, as it reflects the intrinsic interparticle friction and cohesive forces within the granular system. A high angle of repose indicates strong attractive interactions, such as cohesion and adhesion between particles, which in turn reduce powder fluidity and result in poor flowability^32,50^. Representative images of the angle of repose measurements for selected cement samples investigated in this study are presented in Fig. 9.

According to the results presented in Table 5, the angle of repose of the control cement corresponds to the “Very Poor” flow class. In contrast, all samples containing TIPA, DEIPA, and DEG, as well as the P-DEG-0.05 cement, fall into the “Poor” class. All samples incorporating PCE, P-TIPA, and P-DEIPA additives, together with the P-DEG-0.1 cement, are classified within the “Passable” flow class.

The use of GAs reduced the angle of repose for all samples compared with the control cement. In the performance comparison among the GAs themselves, the least improvement was observed with DEG, which reduced the angle of repose by approximately 12–17% relative to the control sample. In contrast, the most pronounced improvement was achieved with the P-TIPA hybrid GA, resulting in a 20–23% reduction.

The enhancement in flow performance observed upon modification of commercial GAs with PCE can be attributed to the high surface adsorption capacity of PCE molecules (Fig. 5), together with the steric hindrance effect induced by the polymer side chains^40,41,49^. However, the dispersion effect observed in dry powdered cement in this study appears to be more limited than the steric hindrance effect provided by PCE in aqueous cement pastes^51^. This finding suggests that, in dry systems, the dominant mechanism governing flow improvement is primarily associated with adsorption-induced electrostatic interactions, rather than steric stabilization.

### Compressive strength

**Fig. 10:**
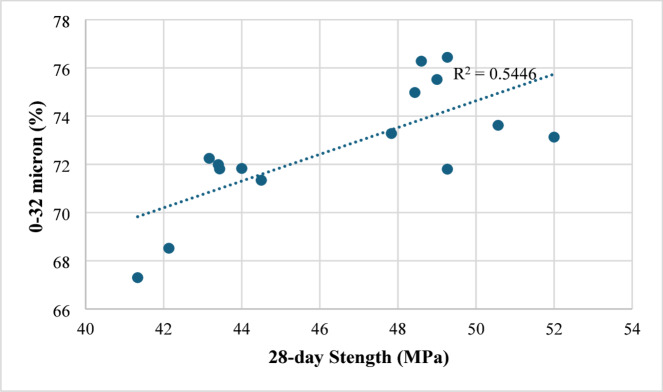
Relationship between compressive strength results and 0–32 micron grain size.

**Fig. 11:**
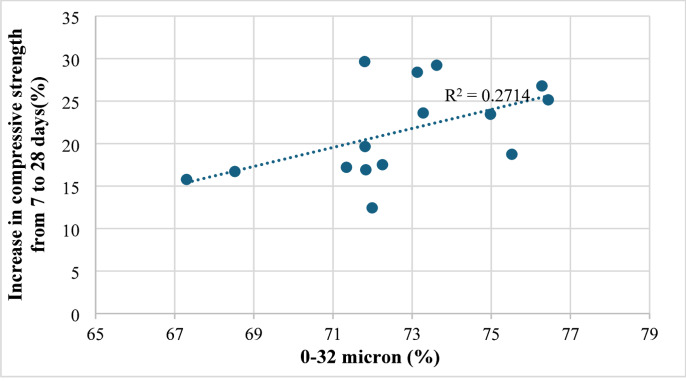
Relationship between the percentage increase in compressive strength from 7 to 28 days and the 0–32 μm particle size fraction.

The 7- and 28-day compressive strength results of the mortar mixtures prepared with the produced cements, together with the d50 value and the fine particle fraction in the 0–32 μm range—both of which are key parameters of the PSD—are presented in Table 6. In addition, the relationship between compressive strength and the 0–32 μm fraction is illustrated in Fig. 10, while Fig. 11 shows the relationship between the strength gain (%) from 7 to 28 days and the 0–32 μm content.

**Table 6:** 7 and 28 day compressive strength results, d50 values, and 0–32 μm particle size fractions of the mortar mixtures.

Mixtures	7-day (Mpa)	Relative Compressive Strength (%)	28-day (Mpa)	Relative Compressive Strength (%)	0–32 mikron	D50 (micron)	Increase in compressive strength from 7 to 28 days (%)
Control	35.70 ± 0.52*	100.00	41.33 ± 0.63	100.00	67.30	19.00	15.78
PCE-0.05	38.00 ± 0.55	106.44	49.27 ± 0.72	119.19	71.80	18.05	29.65
PCE-0.1	38.70 ± 0.57	108.40	47.83 ± 0.69	115.73	73.28	18.73	23.60
TIPA-0.5	39.13 ± 0.60	109.62	50.57 ± 0.74	122.34	73.62	15.90	29.22
TIPA-0.1	40.50 ± 0.62	113.45	52.00 ± 0.78	125.81	73.13	15.40	28.40
P-TIPA-0.5	39.23 ± 0.58	109.90	48.43 ± 0.71	117.18	74.98	14.80	23.45
P-TIPA-0.1	41.27 ± 0.64	115.59	49.00 ± 0.73	118.55	75.52	14.50	18.74
DEIPA-0.05	37.63 ± 0.54	105.42	44.00 ± 0.65	106.45	71.83	16.50	16.92
DEIPA-0.1	37.97 ± 0.56	106.35	44.50 ± 0.67	107.66	71.34	16.30	17.21
P-DEIPA-0.05	39.37 ± 0.59	110.27	49.27 ± 0.72	119.19	76.44	15.20	25.15
P-DEIPA-0.1	38.33 ± 0.56	107.38	48.60 ± 0.70	117.58	76.28	15.00	26.78
DEG-0.05	36.10 ± 0.51	101.12	42.13 ± 0.61	101.94	68.52	18.80	16.71
DEG-0.1	36.30 ± 0.53	101.68	43.43 ± 0.64	105.08	71.81	18.50	19.65
P-DEG-0.05	38.60 ± 0.57	108.12	43.40 ± 0.63	105.00	71.99	18.00	12.44
P-DEG-0.1	36.73 ± 0.52	102.89	43.17 ± 0.62	104.44	72.25	18.10	17.51

*mean (average of three samples) ± SD.

Figure 10 illustrates the relationship between the 28-day compressive strength and the proportion of the 0–32 μm particle size fraction. As shown in the figure, an overall increasing trend is observed, indicating that a higher fine particle fraction generally contributes to improved compressive strength. This behavior can be attributed to the increased specific surface area of ​​finer particles, which is predicted to promote faster hydration kinetics and a denser microstructure. However, the coefficient of determination obtained from the regression analysis (R² = 0.5446) indicates only a moderate correlation between these two parameters.

The moderate R² value suggests that although the fine particle fraction contributes to strength development, it cannot solely explain the variability in compressive strength among the tested mixtures. In cement systems containing GAs, compressive strength is governed not only by PSD but also by chemical and physicochemical interactions introduced by the additives. These include adsorption of GA molecules on clinker surfaces, modification of surface energy, and alterations in hydration kinetics. Consequently, mixtures with similar 0–32 μm fractions may still exhibit different strength levels depending on the type of GA used and its interaction with clinker phases. Therefore, the moderate R² value observed in Fig. 10 suggests that PSD refinement significantly contributes to strength improvement, but additional mechanisms related to GA chemistry and hydration processes can also influence the ultimate compressive strength.

Figure 11 presents the relationship between the percentage increase in compressive strength from 7 to 28 days and the 0–32 μm particle size fraction. In this case, the regression analysis yielded a considerably lower coefficient of determination (R² = 0.2714), indicating a weak correlation between these parameters.

The low R² value implies that the increase in compressive strength between 7 and 28 days is only weakly dependent on the proportion of fine particles. This finding suggests that long-term strength development is more strongly influenced by hydration chemistry and microstructural evolution rather than by particle size alone. In systems containing alkanolamine-based grinding aids such as TIPA and DEIPA, chemical interactions with aluminate and ferrite phases can significantly modify hydration kinetics, promote ettringite formation, and enhance the precipitation of C–S–H at later ages. These chemical effects may vary depending on the specific additive used and its dosage, leading to different strength-gain behaviors even in mixtures with comparable PSD characteristics. Consequently, the weak correlation observed in Fig. 11 suggests that while PSD plays a significant role in early hydration and initial strength formation, strength gain at later ages is primarily influenced by chemical and microstructural factors associated with the grinding aids.

The results summarized in Table 6 indicate that the use of GAs enhances compressive strength irrespective of additive type and dosage. GAs modify the particle size distribution (PSD) of cement during the grinding stage by increasing the 0–32 μm fine fraction and reducing the d50 value, thereby exerting a direct physical influence on hydration kinetics. In addition to these physical effects, it is well established that such additives also play a chemical role in hydration reactions by altering the solution chemistry^31,52,53^. The interplay between these two mechanisms governs both the final compressive strength values and the strength development kinetics observed in GA-containing systems.

For the control cement, a 0–32 μm fraction of 67.3% and a d50 of 19 μm indicate a relatively limited reactive surface area and a broader particle-size distribution compared with GA-containing samples. These physical characteristics resulted in comparatively low compressive strengths of 35.7 MPa at 7 days and 41.3 MPa at 28 days, corresponding to only a 16% increase in strength between 7 and 28 days.

In PCE-modified systems, a marked improvement was observed relative to the control sample in both the 0–32 μm fraction (71.8–73.3%) and the d50 value (~ 18 μm). This led to a 6–8% increase in 7-day strength, with a more pronounced effect at 28 days. However, the observed deceleration in the rate of 28-day strength gain with increasing PCE dosage to 0.1% suggests that PCE, owing to its chemical structure, can partially retard early-age hydration kinetics through complexation with Ca²⁺ ions and surface adsorption^18,41^. Accordingly, the strength enhancement in PCE systems reflects a balance between the physical benefits of PSD refinement and the chemical adsorption effects induced by the additive.

In mixtures containing TIPA, a substantial increase in the fine fraction of the PSD was recorded. The rise in the 0–32 μm fraction to approximately 73% and the reduction in d50 to the 15–16 μm range provided favorable conditions for the formation of a high number of nucleation sites and more intense C–S–H precipitation at early ages. This physical advantage, combined with the chemical effects associated with TIPA’s alkanolamine structure, resulted in the highest 7- and 28-day compressive strength values. In particular, the strengths of 40.5 MPa at 7 days and 52.0 MPa at 28 days achieved in the TIPA-0.1 mixture can be attributed not only to PSD improvement but also to TIPA’s ability to accelerate aluminate and ferrite hydration by increasing the solubility of the C₃A and C₄AF phases^53,55^. Moreover, TIPA regulates ettringite formation and AFt/AFm transformations, ensuring sustained hydration activity at later ages and resulting in a 28% increase in strength between 7 and 28 days.

In P-TIPA hybrid mixtures, the increase in the 0–32 μm fraction to levels of 75% and the decrease of d50 to the lowest values ​​, such as 14.5–14.8 μm, indicate a strong physical synergy in terms of PSD. However, the 28-day strengths of these mixtures lagged behind those of systems containing pure TIPA. This shows that strong surface adsorption of PCE partially suppresses the hydration-accelerating effect of TIPA, and the superior physical PSD advantage does not translate into full chemical synergy at age^42^. Consequently, while PSD largely determines early-age strength in P-TIPA systems, late-age strength results from chemical interactions among the additives. The improvement in PSD in mixtures containing DEIPA remained more limited compared to TIPA, with the 0–32 μm fraction in the 71–72% range and d50 around 16.5 μm. While this physical structure provided a moderate increase in 7-day strengths, the 28-day strengths were only 6–8% above the control sample. Although the alkanolamine structure of DEIPA can interact with aluminate phases, the fact that its activation effect on ferrite phases is not as strong as that of TIPA can be considered one of the main reasons for the limited increase in advanced-age strength^53,56^.

In P-DEIPA hybrid mixtures, significant synergy was observed in both PSD and strength. The increase in the 0–32 μm fraction to 76% and the decrease in d50 to approximately 15 μm provided a physical compactness advantage. Consequently, 28-day strengths reached approximately 49 MPa, with a relative increase of around 19%. The 25% increase in 7–28 day strength, particularly in P-DEIPA mixtures, indicates that the slowdown in PCE-induced hydration kinetics at an early age is compensated for over time, and that the hydration-enhancing effect of DEIPA, especially C_4_AF hydration, becomes more dominant at later ages. In systems containing DEG, PSD, and strength development, a different profile was observed. With DEG, the 0–32 μm fraction increased only to a limited extent (68–72%), while the d50 value remained close to the control sample (~ 18–19 μm). In parallel, the 7 and 28-day strength values ​​also remained close to the control level. The chemical structure of DEG is designed to achieve a more homogeneous PSD by reducing mill friction and agglomeration, rather than directly accelerating hydration reactions^57^. Therefore, the strength improvement in DEG systems was mainly limited to the improvement in physical packing, with a 16–20% increase in strength between 7 and 28 days. Despite the limited improvement in PSD observed in P-DEG hybrid mixtures, the strength increases remained lower than those in PCE-alkanolamine systems. This reveals that the physical advantages provided by PCE are not fully reflected in late-age strength because DEG has limited capacity to chemically activate hydration phases. The results clearly show that strength improvement in cementitious systems cannot be explained solely by PSD, but must also be evaluated in conjunction with solution chemistry and hydration kinetics. Increasing the fine fraction (0–32 μm) accelerates C-S-H formation by increasing the number of nucleation centers at an early age, thus improving the 7-day strength. Conversely, reducing the coarse tail fraction (> 60 μm) and the d90 value significantly affects the 28-day strength by decreasing the amount of inert nuclei that do not react at later ages.

The primary function of PCE is to homogenize the cement particle PSD and reduce the coarse particle fraction by suppressing agglomeration during grinding. This physical effect is particularly effective in enhancing long-term strength. However, the adsorption of PCE onto cement particle surfaces and its tendency to form complexes with Ca²⁺ ions^18,31,42^ can retard early-age hydration kinetics, and this effect becomes more pronounced as the dosage increases. Accordingly, optimum performance in PCE-based systems is governed by the balance established between physical gains and chemical interactions.

Alkanolamine-derivative GAs such as TIPA and DEIPA not only improve the PSD but also directly influence hydration chemistry. These admixtures form complexes with Fe³⁺ and Al³⁺ ions, thereby increasing the solubility of ferrite and aluminate phases, regulating ettringite formation and AFt/AFm transformations, and sustaining their effectiveness during the later stages of hydration^35,50^. Consequently, very high later-age strengths were achieved in systems containing TIPA.

In hybrid systems consisting of physical mixtures of PCE and alkanolamines, a pronounced synergy was obtained in terms of PSD refinement; however, the final strength values varied depending on the nature of the chemical interactions between the additives. For example, in the P-DEIPA-0.05 mixture, physical and chemical effects complemented each other, whereas in the P-DEIPA-0.1 mixture, early-age strength decreased due to surface adsorption and kinetic suppression, but high later-age strength was still achieved owing to the packing advantage provided by PSD optimization. These findings demonstrate that, in the design of hybrid grinding-facilitator admixtures, PSD optimization alone is insufficient; instead, defining a composition and dosage range compatible with solution chemistry and hydration kinetics is critical.

Overall, this study reveals that the mechanisms by which grinding-facilitator admixtures govern cement performance are inherently multidimensional. Both physical effects (PSD refinement and particle packing) and chemical effects (ion complexation and hydration catalysis) must be considered simultaneously. In particular, it is evident that PCE-alkanolamine combinations, when properly designed, have the potential to optimize both early- and later-age strength. However, realizing this potential requires establishing a balanced network of physical–chemical interactions.

## Conclusion

Based on the materials used and experiments conducted within the scope of this study, the following results were obtained, summarized below:

- Hybrid GAs were shown to significantly reduce grinding energy compared with single-component GAs. In particular, the P-DEIPA mixture achieved the highest efficiency, providing an energy saving of 14.4%. This superior performance is attributed to the synergistic interaction between the steric hindrance and agglomeration-control effects of PCE and the surface energy–reducing and fracture-facilitating mechanisms of alkanolamines.
- Particle size distribution analyses demonstrated that hybrid systems achieved both the highest fine particle content (76.4% for P-DEIPA) and the most homogeneous distributions. The most pronounced improvement in powder fluidity was observed with the P-TIPA mixture, highlighting the dominant role of PCE adsorption-induced surface modification in dry powder systems.
- It was established that compressive strength performance depends not only on physical particle size distribution but also on the influence of GAs on hydration chemistry. TIPA yielded the highest early- and later-age strengths (52.0 MPa at 28 days) through the combined action of physical refinement and chemical acceleration, whereas the performance of hybrid systems varied according to the physical–chemical balance between the GA components.
- Physical mixtures of PCE with alkanolamine- and glycol-derivative GAs were shown to provide a superior alternative to conventional single-component GAs by simultaneously enhancing cement grinding efficiency, powder flow properties, and ultimate strength performance.

## Data Availability

All data generated or analyzed during this study are included in this published article.
